# Evidence to support magnetic resonance conditional labelling of all pacemaker and defibrillator leads in patients with cardiac implantable electronic devices

**DOI:** 10.1093/eurheartj/ehab350

**Published:** 2021-08-26

**Authors:** Anish N Bhuva, Russell Moralee, Tamara Brunker, Karen Lascelles, Lizette Cash, Kush P Patel, Martin Lowe, Neha Sekhri, Francisco Alpendurada, Dudley J Pennell, Richard Schilling, Pier D Lambiase, Anthony Chow, James C Moon, Harold Litt, A John Baksi, Charlotte H Manisty

**Affiliations:** Department of Cardiology, Barts Heart Centre, Barts Health NHS Trust, London, EC1A 7BE, UK; Institute for Cardiovascular Science, University College London, London, WC1E 6HX, UK; Health Data Research UK, University College London, London, UK; Department of Cardiology, Barts Heart Centre, Barts Health NHS Trust, London, EC1A 7BE, UK; Department of Radiology, Division of Cardiovascular Medicine, Perelman School of Medicine, University of Pennsylvania, Philadelphia, PA, USA; Cardiovascular Magnetic Resonance Unit, Royal Brompton & Harefield NHS Foundation Trust and National Heart and Lung Institute, Imperial College, London, SW3 6NP, UK; Department of Cardiology, Barts Heart Centre, Barts Health NHS Trust, London, EC1A 7BE, UK; Department of Cardiology, Barts Heart Centre, Barts Health NHS Trust, London, EC1A 7BE, UK; Department of Cardiology, Barts Heart Centre, Barts Health NHS Trust, London, EC1A 7BE, UK; Department of Cardiology, Barts Heart Centre, Barts Health NHS Trust, London, EC1A 7BE, UK; Cardiovascular Magnetic Resonance Unit, Royal Brompton & Harefield NHS Foundation Trust and National Heart and Lung Institute, Imperial College, London, SW3 6NP, UK; Cardiovascular Magnetic Resonance Unit, Royal Brompton & Harefield NHS Foundation Trust and National Heart and Lung Institute, Imperial College, London, SW3 6NP, UK; Department of Cardiology, Barts Heart Centre, Barts Health NHS Trust, London, EC1A 7BE, UK; Department of Cardiology, Barts Heart Centre, Barts Health NHS Trust, London, EC1A 7BE, UK; Institute for Cardiovascular Science, University College London, London, WC1E 6HX, UK; Department of Cardiology, Barts Heart Centre, Barts Health NHS Trust, London, EC1A 7BE, UK; Department of Cardiology, Barts Heart Centre, Barts Health NHS Trust, London, EC1A 7BE, UK; Institute for Cardiovascular Science, University College London, London, WC1E 6HX, UK; Department of Radiology, Division of Cardiovascular Medicine, Perelman School of Medicine, University of Pennsylvania, Philadelphia, PA, USA; Cardiovascular Magnetic Resonance Unit, Royal Brompton & Harefield NHS Foundation Trust and National Heart and Lung Institute, Imperial College, London, SW3 6NP, UK; Department of Cardiology, Barts Heart Centre, Barts Health NHS Trust, London, EC1A 7BE, UK; Institute for Cardiovascular Science, University College London, London, WC1E 6HX, UK

**Keywords:** Pacemaker, Defibrillator, Magnetic resonance imaging

## Abstract

**Aims:**

Many cardiac pacemakers and defibrillators are not approved by regulators for magnetic resonance imaging (MRI). Even following generator exchange to an approved magnetic resonance (MR)-conditional model, many systems remain classified ‘non-MR conditional’ due to the leads. This classification makes patient access to MRI challenging, but there is no evidence of increased clinical risk. We compared the effect of MRI on non-MR conditional and MR-conditional pacemaker and defibrillator leads.

**Methods and results:**

Patients undergoing clinical 1.5T MRI with pacemakers and defibrillators in three centres over 5 years were included. Magnetic resonance imaging protocols were similar for MR-conditional and non-MR conditional systems. Devices were interrogated pre- and immediately post-scan, and at follow-up, and adverse clinical events recorded. Lead parameter changes peri-scan were stratified by MR-conditional labelling. A total of 1148 MRI examinations were performed in 970 patients (54% non-MR conditional systems, 39% defibrillators, 15% pacing-dependent) with 2268 leads. There were no lead-related adverse clinical events, and no clinically significant immediate or late lead parameter changes following MRI in either MR-conditional or non-MR conditional leads. Small reductions in atrial and right ventricular sensed amplitudes and impedances were similar between groups, with no difference in the proportion of leads with parameter changes greater than pre-defined thresholds (7.1%, 95% confidence interval: 6.1–8.3).

**Conclusions:**

There was no increased risk of MRI in patients with non-MR conditional pacemaker or defibrillator leads when following recommended protocols. Standardizing MR conditions for all leads would significantly improve access to MRI by enabling patients to be scanned in non-specialist centres, with no discernible incremental risk.

## Graphical Abstract

**Graphical Abstract:**
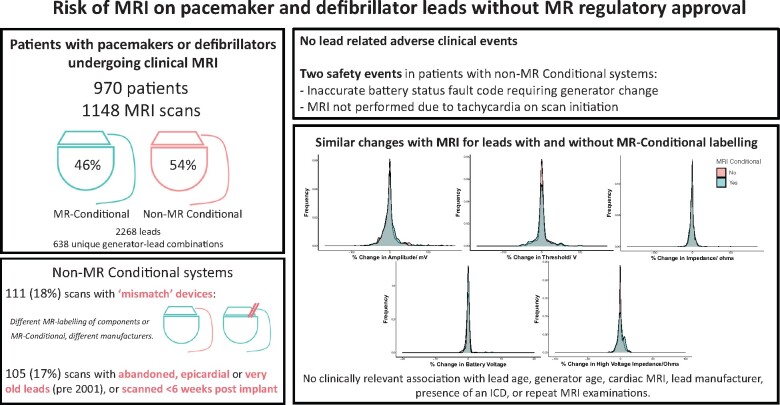
Clinicians can safely perform magnetic resonance imaging in non-specialist centres for patients with pacemakers and defibrillators if the generator is MR-conditional, irrespective of the magnetic resonance safety labelling of the attached leads, by following standardized protocols adapted from fully MR-conditional systems and excluding other high-risk scenarios.

**See the editorial comment for this article ‘Reconsidering safety and reducing barriers to MRI in patients with cardiac implantable electronic devices’, by Chiara Bucciarelli-Ducci and Panos Vardas, https://doi.org/10.1093/eurheartj/ehab469.**

## Introduction

Access to magnetic resonance imaging (MRI) for patients with cardiac implantable electronic devices (CIEDs—permanent pacemakers, and implantable cardioverter-defibrillators, ICDs) has been improved by the development of magnetic resonance (MR)-conditional devices, designed to reduce both the risk and technical and logistic burdens of scanning.^1,2^ Despite this and whilst the majority of CIED patients will need MRI in their lifetime, they are less likely to be referred and scans are frequently delayed or inappropriately denied.^3–5^

Unfortunately, the majority of CIEDs *in situ* worldwide have not received regulatory approval (termed ‘non-MR conditional’).^6^ Recent data have shown the risk of scanning patients with these devices is low when following strict protocols,^6–8^ and MRI is now recommended where clinical indications are robust. Very few institutions, however, will provide MRI to patients with non-MR conditional devices because of persistent safety concerns and the additional regulatory requirements mandated in the recommended protocols.^3,9–11^ This leads to even greater barriers to accessing MRI for this group of patients, particularly in scenarios considered the highest risk, such as in the presence of an abandoned lead for which there is less published safety data.^11^

Although exchanging a non-MR conditional CIED generator for an MR-conditional replacement is relatively straightforward, leads are generally implanted permanently as lead extraction carries considerable risk (0.4–2% procedural mortality).^12^ Cardiac implantable electronic device system MR labelling is based on the complete system (generator and leads combination) and given that there are more than 3.5 million non-MR conditional leads implanted in US citizens alone,^6^ this will remain a problem for many years to come.

*In vitro* experiments scanning older leads using historical protocols highlighted a risk of lead-related tissue heating or lead failure;^13–16^ however, there have been no reported clinical adverse events.^17^ We hypothesized that there is no increased risk of MRI in patients with non-MR conditional leads compared with those with leads labelled as MR-conditional. At 1.5T (1.5T), we compared the frequency of clinical MRI safety events and lead parameter changes in a dedicated multi-centre clinical device MRI cohort, stratified by lead MR-conditional labelling.

## Methods

### Study design, ethical approval statement

A multi-centre study of patients with a pacemaker or ICD undergoing clinically indicated MRI at 1.5T. The research protocol was approved by local institutional review boards in the USA and UK and a local committee of the National Research Ethics Service in the UK (14379/001). Written, informed consent for MRI was obtained from all patients with non-MR conditional devices. The study complied with the Declaration of Helsinki. The funders did not have any role in study design, analysis, or interpretation of results.

### Study population

Patients with pacemakers and defibrillators undergoing MRI between 2014 and 2019 across three hospital sites in UK and the USA were included. Patients with both non-MR conditional and MR-conditional devices were recruited prospectively at Barts Heart Centre (London, UK) and retrospectively included from the Royal Brompton Hospital (London, UK). Only patients with non-MR conditional devices with insurance coverage, typically aged over 65 years, provided by the US Centers for Medicare and Medicaid Services (CMS), were recruited prospectively at the Hospital of the University of Pennsylvania (Philadelphia, PA, USA) under a ‘coverage with evidence determination’ protocol, allowing reimbursement for MRI (NCT02513056).

All MRI scans were performed according to international guidance following manufacturer recommendations where available, summarized in *Table 1*.^18^ Local institutional protocols have been described previously.^19–21^ Unlike previous studies,^6,7^ patients were not excluded if they had an abandoned lead, permanent epicardial lead, manufacturer date before 2001,^13^ recent implantation, or a battery at the elective replacement interval/time, or deactivated systems, but were categorized as non-MR conditional (Supplementary material online, *Methods*)^18^

**Table 1:** Summary protocol for patients with cardiac implantable electronic devices undergoing magnetic resonance imaging

**Before scan**
Identification of MR-conditional labelling of each component and system.
Identification of high-risk risk scenarios (fractured, epicardial, abandoned leads; recent implantation; battery at ERI; deactivated systems; lead parameters outside manufacturer recommendations, other implants present).
For patients with non-MR conditional devices—Discussion of risk-benefit including informed written patient consent, confirmation from the referrer that the scan will change clinical management and that no alternative imaging modalities can answer the clinical question.
Device interrogation and appropriate programming, following manufacturer protocols and using MR mode software where available.
Appropriate MRI protocol prescribed.
**During scan**
Scan in normal operating mode.
Monitor ECG, pulse oximetry, and maintain verbal contact.
Personnel with ability to perform advanced cardiac life support available as per institutional protocol.
External defibrillator with transcutaneous pacing capacity available.
**After scan**
Device interrogation and appropriate reprogramming to usual settings.
Clinic follow-up arranged as per institution protocol.

All MRI scans were performed according to international guidance following manufacturer recommendations where available.^18^

ECG, electrocardiogram ERI, elective replacement indicator; MR, magnetic resonance; MRI, magnetic resonance imaging.

### Cardiac implantable electronic device interrogation and programming

Patients underwent CIED interrogation and reprogramming immediately before MRI in accordance with guideline recommendations.^18^ In brief, an asynchronous pacing mode was programmed at high output for pacing-dependent patients, otherwise, a non-pacing or inhibited mode was programmed. Anti-tachycardia detection and therapies were disabled for ICDs. Lead parameters (sensed amplitude, capture threshold, and impedance) that indicate interaction with surrounding tissue, and battery status, were measured and recorded.

### Magnetic resonance imaging protocol

All studies were performed at one of five magnets at 1.5T (Aera, Avanto, Avanto Fit, Espree; Siemens Healthcare, Erlangen, Germany) in normal operating mode (whole-body-specific absorption rate restricted to 2 W/kg). Otherwise, standard imaging protocols were used for the clinical indication, with no adaptation according to CIED MR-conditionality. Patients with MR-conditional CIEDs requiring MRI scans within isocenter exclusions zones stated by the manufacturer were scanned as an off-label scan, but were recorded as an MRI in a patient with an MR-conditional CIED for this analysis. Patients were monitored using verbal contact, continuous pulse oximetry, and electrocardiogram monitoring by appropriately trained staff.

### Cardiac implantable electronic device reprogramming and follow-up

Immediately after MRI, repeat CIED interrogation was performed and recorded, with CIED programming restored to the original pre-MRI settings. Late follow-up CIED interrogations were scheduled according to standard clinical protocols, and reports were available for patients followed up locally. If there were concerns regarding CIED parameter changes immediately post-MRI, earlier follow-up was arranged at clinician’s discretion.

### Primary and secondary endpoints

Endpoints for MRI-related clinical safety events and CIED parameter changes were defined prospectively.

Clinical safety events recorded were death, lead failure, sustained symptomatic or life-threatening arrhythmia, complete or partial electrical reset, generator malfunction, inappropriate inhibition of pacing, or inappropriate anti-tachycardia therapies. Lead failure was defined as the need for lead replacement or revision. Clinical safety events were adjudicated by a panel of senior investigators (C.H.M./H.L./A.J.B.).

It is recognized that there are minor temporal fluctuations in lead parameters, even for MR-conditional leads.^22^ We pre-defined thresholds for significant changes attributable to MRI (outside the range of normal measurement fluctuation) based on previously published data (details provided in Supplementary material online, *Methods*).^7,23^ Late follow-up CIED interrogations were used to assess the longevity of lead parameter or battery voltage changes.

Individual device components (leads and generators) were defined as MR-conditional if they had been independently approved as part of an MR-conditional system, adjudicated at the time of analysis. In order to assess the potential impact of the MR-conditionality of the generator, a sensitivity analysis was performed with analysis stratified by complete CIED system MR-conditional labelling. Cardiac implantable electronic device systems can also be ‘mismatched’ where either one or more components of an otherwise MR-conditional system is non-MR conditional, or where MR-conditional components from different manufacturers are combined.

### Statistics

Data were analysed in R (R Foundation, Vienna, Austria) using RStudio Server version 1.0.153 (RStudio Inc., Boston, MA, USA). Continuous lead parameter variables are expressed as median (interquartile range), and changes presented as a 95% non-parametric confidence interval (CI) with continuity correction and the median change. Percentage differences were used for comparisons to account for baseline differences in absolute values. Linear regression was used to investigate whether MR-conditional and non-MR conditional leads demonstrated similar influences (lead and generator age, thoracic or cardiac MRI, lead manufacturer, presence of an ICD, and repeat MRI examinations) on lead-tissue interaction whilst in an MR environment (Supplementary material online, *Methods*). All tests were two-tailed. Control for the false discovery rate was not performed to minimize the probability of a type II error.

## Results

### Baseline characteristics

Overall, 1148 clinically indicated MRI examinations [506 (44%) cardiac] were performed in 970 patients (15% pacing-dependent) with a total of 2268 leads. Clinical safety events were recorded for all patients. Lead parameters from 99 examinations were not available because of battery depletion in a deactivated CIED (*n* = 1), or incomplete lead documentation (*n* = 98). A total of 1049 CIED systems with 2088 leads in 889 patients were therefore analysed for changes in lead parameters (*Table 2*). In total, 615 (54%) CIEDs were non-MR conditional systems; 703 (61%) pacemakers; and 445 (39%) ICDs or cardiac resynchronization therapy-defibrillators. There were one (14%), two (72%), or three (14%) generator-attached leads in each system. Forty patients had an abandoned lead (one with two abandoned leads, one also with a subcutaneous array). Three patients had surgically implanted permanent epicardial pacing leads. Nine patients had subcutaneous ICDs. Thirty-one (3%) systems were recently implanted <6 weeks before MRI.

**Table 2:** Baseline patient and cardiac implantable electronic device characteristics

Baseline characteristics	MR-conditional system		Non-MR conditional system		*P*
No. of scans	533		615		
No. of patients	462		509		
**Patient characteristics**					
Age (years), median (IQR)	65	(50, 75)	73	(65, 79)	<0.001
Male sex	369	(69%)	430	(70%)	0.6305
Hospitalized inpatient	39	(7%)	31	(5%)	0.1079
Previous MRI with a CIED	71	(13%)	106	(17%)	0.0973
Pacing-dependent	70	(13%)	98	(16%)	0.2092
**MRI examination**					
Cardiac	321	(54%)	185	(27%)	<0.001
Spine	122	(20%)	158	(23%)	
Head	93	(16%)	202	(30%)	
Abdomen or pelvis	46	(8%)	91	(14%)	
Extremity or joint	9	(1%)	36	(5%)	
Other	6	(1%)	3	(1%)	
**Device characteristics**					
PPM	332	(62%)	330	(54%)	0.0352
ICD	149	(28%)	168	(27%)	
CRT-P	15	(3%)	26	(4%)	
CRT-D	37	(7%)	91	(15%)	
**Pulse generator characteristics**					
MR-conditional	533	(100%)	122	(20%)	–
Age (years), median (IQR)	1	(1, 2)	4	(2, 6)	<0.001
Number older than 10 years	0	(0%)	13	(2%)	
Pulse generator manufacturer					
Boston Scientific	139	(26%)	183	(30%)	<0.001
Abbott	44	(8%)	151	(25%)	
Biotronik	64	(12%)	21	(3%)	
Medtronic	282	(53%)	241	(39%)	
Sorin	4	(1%)	17	(3%)	
**Lead characteristics**	**MR-conditional lead**		**Non-MR conditional lead**		
RA lead	739	(43%)	228	(42%)	0.0746
RV lead	559	(32%)	177	(32%)	
HV (ICD) lead	312	(18%)	88	(16%)	
LV lead	112	(7%)	53	(10%)	
Age (years)	2	(1, 4)	8	(4, 12)	<0.001
No. greater than 10 years old	7	0 (4%)	168	(31%)	—
Abandoned leads		—	40	(7%)	—

Magnetic resonance conditionality stratification is by complete CIED system classification except for lead numbers, which are stratified by individual lead MR-conditionality. Pacesetter leads were labelled as Abbott (St Jude Medical); Intermedics as Boston Scientific; ELA as Sorin; and Vitatron as Medtronic. One patient underwent lead extraction and replacement between MRI scans changing magnetic resonance conditionality of the device, so total number of unique patients is 970. Some patients had multiple body part examinations per individual scan.

CIED, cardiac implantable electronic device; CRT-P/D, cardiac resynchronization therapy-pacemaker/defibrillator; HV, high voltage; ICD, implantable cardioverter-defibrillator; IQR, interquartile range; MRI, magnetic resonance imaging; PPM, permanent pacemaker.

Non-MR conditional systems had been implanted for longer duration with a higher proportion of ICDs than MR-conditional systems (*Table 2*). A total of 168 (31%) non-MR conditional leads (earliest implant date 1985), and 70 (4%) MR-conditional leads were implanted for over 10 years. Thirty-one (6%) non-MR conditional leads were implanted before 2001. Overall, 246 generator models, 210 lead models and 638 unique generator-lead combinations were studied (detailed in Supplementary material online, *Table S1* and *S2*). Patients with non-MR conditional systems were older, more commonly hospitalized inpatients, and more frequently underwent neurological, and less frequently cardiac scans than patients with MR-conditional systems.

### Clinical safety events

There were no deaths or lead failures, no complete or partial electrical resets, no inappropriate inhibition of pacing and no inappropriate anti-tachycardia therapies during or immediately after MRI (combined clinical safety endpoint 95% CI 0, 0.3%).

In patients with non-MR conditional CIEDs, there were two safety events. One patient with a non-MR conditional dual-chamber ICD and non-MR conditional leads implanted in 2012 required urgent generator replacement following a fault code for inaccurate battery status estimation 1 week post-MRI. The generator was already under a manufacturer advisory, and the fault was reported to the manufacturer. One patient with a redundant inactive (battery completely depleted, non-interrogable) non-MR conditional dual-chamber ICD and non-MR conditional leads implanted in 1999 developed a tachycardia and chest tightness on initiating the scan necessitating evacuation, with immediate normalization of heart rhythm and symptoms. The scan was re-attempted subsequently with repeat sequence of events, leading to scan abandonment. In patients with MR-conditional CIEDs, there were eight ICD generator audible alarm failures after MRI. This is a manufacturer recognized issue and required no further action.

### Lead parameters changes after magnetic resonance imaging

**Figure 1:**
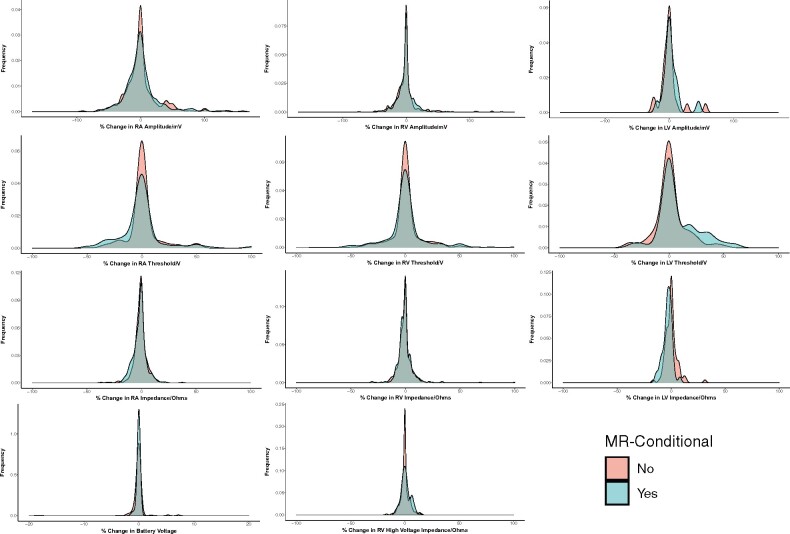
Comparison of changes in lead parameters immediately after magnetic resonance imaging, stratified by magnetic resonance conditionality of individual leads. Density plots x-axis represents the percentage change (post minus pre) in the lead parameter. All changes after magnetic resonance imaging are similar between MR-conditional and non-MR-conditional leads except for a slightly greater decrease in the left ventricular lead threshold of magnetic resonance-conditional leads. Left, centre, and right columns refer to right atrial (RA), right ventricular (RV), and left ventricular (LV) leads, respectively, for the top three rows. First row: Changes in lead sensing; Second row: Changes in lead threshold; Third row: Changes in lead impedance; Fourth row: Changes in battery voltage and right ventricular high voltage impedance (distal coil).

There were no lead parameter changes following MRI scans requiring modification to device programming. Across all patients, capture thresholds did not change after MRI, although there were small reductions in right atrial and right ventricular lead sensed amplitudes, and small reductions in the impedance of all leads (*Table 3*). When stratified by lead MR-conditionality, the changes were no greater with non-MR conditional leads, with less change in left ventricular lead capture threshold than with MR-conditional leads (*Figure 1*).

**Table 3:** Changes in lead parameters (post minus pre) after magnetic resonance imaging scanning, stratified by magnetic resonance conditionality of individual leads

Lead variable	MR-conditional leads								Non-MR conditional leads								*P*
	*N*	Pre	Post			Change (95% CI)		*P*	*N*	Pre		Post		Change (95% CI)		*P*	(between groups)
**Right atrial**																	
Amplitude (mV)	630	3.4	(2.2, 4.9)	3.3	(2.1, 4.8)	−0.1	(−0.05, −0.18)	**0.002**	177	2.7	(1.6, 4.3)	2.8	(1.5, 4)	−0.1	(0.05, −0.2)	0.304	0.350
Threshold (V)	602	0.7	(0.5, 0.75)	0.7	(0.5, 0.75)	0.0	(0, −0.07)	0.113	178	0.75	(0.5, 1)	0.75	(0.6, 1)	0.0	(0.1, 0)	0.169	0.124
Impedance (Ω)	664	490	(437 580)	486.5	(432 569)	−11.0	(−9, −14)	**<0.001**	188	480	(399 581)	469	(400 559)	−7.5	(−2.5, −12)	**0.004**	0.198
**Right ventricular**																	
Amplitude (mV)	673	11.8	(7.8, 15.8)	11.4	(8, 16)	−0.3	(−0.25, −0.45)	**<0.001**	199	10.9	(6, 12)	10.25	(6, 12)	−0.4	(−0.2, −0.7)	**<0.001**	0.666
Threshold (V)	785	0.75	(0.6, 1)	0.75	(0.55, 1)	0.0	(0, −0.03)	0.525	235	0.9	(0.75, 1.25)	0.9	(0.75, 1.25)	0.0	(0.08, −0.02)	0.468	0.567
Impedance (Ω)	780	494	(436 605)	494	(430 590)	−9.5	(−6, −11)	**<0.001**	238	540	(441 655)	536	(440 637)	−8.0	(−4, −12)	**<0.001**	0.461
HV impedance (Ω)	282	65	(51, 77)	66.5	(50, 77)	0.5	(1, 0)	0.178	84	51	(47, 62)	51.5	(48, 61)	−0.5	(0.5, −1)	0.390	0.453
**Left ventricular**																	
Amplitude (mV)	26	17.8	(10, 25)	16.85	(11, 25)	0.1	(0.95, −0.9)	0.887	17	14.1	(10, 18)	15	(10, 18)	−0.2	(0.51, −0.81)	0.629	0.617
Threshold (V)	96	1.05	(0.75, 1.5)	1.1	(0.75, 1.5)	0.1	(0.2, 0.02)	**0.015**	44	1.00	(0.75, 1.5)	1.00	(0.75, 1.25)	−0.1	(0.03, −0.35)	0.123	**0.003**
Impedance (Ω)	92	716	(563 852)	703	(549 858)	−15.5	(−6, −25)	**0.002**	45	722	(494 977)	689	(523 955)	−13.0	(0, −26)	0.058	0.266

Comparisons between MR-conditional and non-MR conditional lead groups are made as comparisons of percentage change to avoid baseline differences in absolute measurements. The number of lead comparisons is smaller than the number of total patients and depends on the number of leads implanted, variable underlying rhythm, variable pulse width, device, and the presence of atrial fibrillation. In these cases, there was no clinically significant change on interrogation. *P*-values are for pre- vs. post-comparisons and for the comparison of changes post-MRI between MR-conditional and non-MR conditional leads (between groups). Statistically significant comparisons shown in bold (p<0.05).

MR, magnetic resonance; MRI, magnetic resonance imaging.

**Figure 2:**
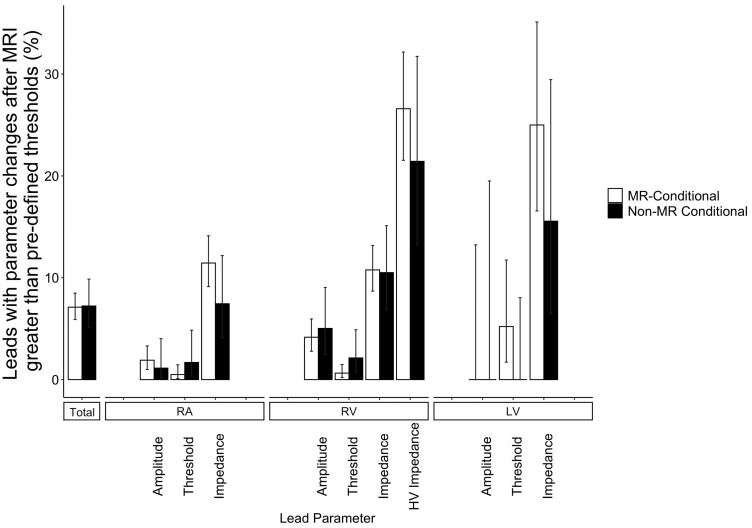
Frequency of leads exceeding normal expected variability after magnetic resonance imaging, stratified by lead magnetic resonance-conditional labelling. Total frequencies are statistically similar for MR-conditional and non-MR-conditional leads. Pre-defined thresholds for measured changes considered greater than normal measurement fluctuation and attributable to magnetic resonance imaging are based on published data.^23^ Cut-off values were: a decrease in sensed P-wave amplitude ≥50%; a decrease in sensed R-wave amplitude ≥25%; an increase in capture threshold ≥0.5 V; an absolute change in pacing lead impedance ≥50 Ω; an absolute change in high voltage lead impedance ≥3 Ω; a decrease in battery voltage ≥0.04 V.^7^ Error bars represent estimated 95% confidence intervals.

Using pre-defined thresholds for changes in lead parameters, the proportion of leads with changes exceeding expected normal variability were no different between MR-conditional and non-MR conditional leads [7.1%, (95% CI 5.9, 8.5) vs. 7.2% (95% CI 5.1, 9.9), *P* = 0.93] (*Figure 2*). There were no greater differences for patients with ICDs (*n* = 445 scans, 39%, Supplementary material online, *Table S3*), patients with generator-lead ‘mismatch’ in MR-conditionality (*n* = 111 scans, 18% of non-MR conditional systems, Supplementary material online, *Table S4*), or when stratified by overall CIED system (rather than individual lead) MR-conditionality (Supplementary material online, *Table S5*).

Late follow-up was available from 740 (64%) MRI examinations in 629 (65%) patients at 72 (29–150) days (Supplementary material online, *Table S6*). There were no differences in changes to lead parameters at late follow-up when stratified by MR-conditionality (Supplementary material online, *Table S7* and *S8*).

### Influences on lead parameter changes following magnetic resonance imaging

Several device and scan characteristics had small but statistically significant associations with lead parameter changes with MRI; however, these explained only a small amount of the total variation in lead parameter changes (<10%) (Supplementary material online, *Table S9*). There were no clinically relevant differences in the device (including generator and lead age) and scan characteristics (including cardiac MRI) associated with MR-conditional and non-MR conditional lead parameter changes after MRI.

### Battery voltage changes after magnetic resonance imaging

Across all patients, there was no change in battery voltage immediately after MRI (0.00, 95% CI −0.005, 0.005 V). Ten CIEDs demonstrated a battery voltage decrease of at least 0.04 V immediately after MRI (nine non-MR conditional, two pacemakers, eight ICDs, implanted between 2011 and 2015). At follow-up, battery voltage had recovered to baseline values (*n* = 8) or was stable (*n* = 2). One patient had previously undergone two other MRI scans without changes in battery voltage.

## Discussion

These data show that MRI in patients with cardiac pacemakers and ICDs is safe provided appropriate protocols are followed, with no excess risk in patients with leads that do not have regulatory MRI approval (‘non-MR conditional’). There were no lead failures, and the incidence of small (clinically insignificant) changes in lead parameters was similar in patients with non-MR conditional and MR-conditional leads, even for higher perceived risk groups (defibrillators, older components, thoracic MRI, repeat scans, pacing-dependent). These results reflect a broad range of real-world patients (*n* = 970), scans (*n* = 1148), and cardiac devices (5 manufacturers, 241 generators and 210 lead models, 683 unique lead-generator combinations) over 5 years and three centres in two continents. Given these data and the quantified harm from barriers to undergoing MRI, we believe that MR conditions can be standardized for all leads to permit MRI using protocols for current MR-conditional CIEDs (*Graphical abstract*).

The development of MR-conditional pacemakers and ICDs has enabled patients to undergo MRI in accordance with manufacturer guidance at extremely low risk. The majority of patients worldwide, however, have non-MR conditional devices and have even greater difficulty accessing MRI despite recent safety data.^3–6^ Exchanging a non-MR conditional generator for an MR-conditional one does not remove the problem—the presence of the pre-existing non-MR conditional leads currently renders most new generator-lead combinations non-MR conditional. This constituted 18% of patients in this registry with non-MR conditional CIEDs, but because patients are likely to have the same non-MR conditional leads over their lifespan,^24^ this population is expected to grow as generators are replaced. Whilst these patients can undergo MRI, this is mostly only offered in specialist centres, and so the majority of patients still report challenges accessing MRI for urgent diagnoses or cancer care.^3–5,10^ The findings of this study may be most readily translated by providing MRI to patients with such ‘mismatch’ CIED components outside of specialist centres.

Risks of MRI to patients with cardiac devices can be related to the generator (hardware or software damage), lead failure or lead-related tissue heating. Despite *in vitro* evidence of lead-related heating prompting re-design in MR-conditional leads, no adverse patient events have previously been reported with non-MR conditional leads when adhering to appropriate protocols.^8,16,17^ The current study confirmed this with no clinically significant changes in lead parameters with MRI and no difference in the incidence of minor changes between MR-conditional and non-MR conditional leads. All changes after MRI were small (within normal expected variability),^1,17^ and the confidence intervals narrow, suggesting the possibility of any larger, more clinically significant, change is unlikely. The influence on lead parameters of higher perceived risk groups demonstrated no associations that should impact on clinical decision-making.^25^

These data suggest that when appropriate protocols are followed, lead-related tissue heating or lead failure do not pose a clinical risk, and that current MR safety labelling of leads has no detectable effect on the clinical risk profiles of undergoing MRI. Although retrospective re-labelling of leads as MR-conditional has been performed, the number of lead-generator combinations in this study (*n* = 638) illustrates the challenges of formal regulatory testing of all possible system configurations. The decision standard is of comparison to current practice (MRI for patients with MR-conditional CIEDs) and of demonstrating net benefit against opportunity costs of inaction, namely delay or second-line imaging, invasive testing, or no investigation.^25^ The lack of safety concerns despite systematic data collection of sensitive lead and generator parameters prompts re-assessment of MR-conditional labelling for all leads and, together with recent studies, support reducing barriers to providing MRI services for patients with CIEDs.^6,7^

Whilst risk is low when following strict safety protocols, it appears to be driven clinically by factors related to the generator and not the leads. There was a single clinical safety event across all patients with active CIEDs and was secondary to an inaccurate battery status estimation fault code. This has occurred in 2.3% of patients with similar generator models unrelated to MRI.^26^ This registry also included patients not recruited to other large registries due to abandoned leads, subcutaneous arrays, permanent epicardial leads, deactivated CIEDs, and CIEDs implanted before 2001.^6,7^ Implantable cardioverter-defibrillators implanted before 2001 have demonstrated higher risk of MRI,^13^ and this is consistent with the observation of symptomatic arrhythmia in one patient with a deactivated ICD implanted in 1999. Electrical reset has previously been described in generators implanted before 2006, but was not observed in this cohort, likely due to the low number of patients with these systems reflecting the very small (and rapidly declining) numbers of patients with these older models *in situ*.^17^ Workflows comprising appropriate cardiac device interrogation and programming, specific MRI scanning restrictions and patient monitoring remain necessary.

### Study limitations

The study did not examine all possible generator-lead combinations for either MR-conditional or non-MR conditional devices. Scans were limited to different 1.5 systems from a single MRI manufacturer, and all scans were low specific absorption rate device protocols although details of specific MR energies were not available. Some rarer scenarios (abandoned lead or inactive generators) were not sufficiently represented. Older components that may have higher risk of undergoing MRI were relatively under-represented, but constitute an ever decreasing proportion of active implants. Whilst most CIED complications occur around the time of MRI, we cannot be certain that later complications occurred or were not captured in patients without follow-up.

## Conclusion

There is no incremental risk of either clinical safety events or early changes to device or lead performance from 1.5T MRI for patients with non-MR conditional pacemaker or defibrillator leads compared with those labelled MR-conditional, when approved protocols are followed. This suggests that MR conditions can be standardized for CIED leads in the majority of cases, with the advantage of increased access to MRI for patients with CIEDs outside of specialist centres. Further research is required to assess whether the results are generalizable to the presence of abandoned leads or other scenarios considered higher risk.

## Supplementary material

Supplementary material is available at *European Heart Journal* online.

## Funding

A.N.B. is supported by a research and innovations grants from the British Heart Foundation (FS/16/46/32187, HFHF_016) and a research grant from Abbott Laboratories. J.C.M., P.D.L., and C.H.M. are directly and indirectly supported by the NIHR Biomedical Research Centres at University College London Hospital and Barts Health NHS Trusts. The authors are solely responsible for the concept, design, and conduct of the study.

**Conflict of interest:** A.N.B. and C.H.M have received funding from Abbott Laboratories. D.J.P. has received research funding from Siemens. A.C. has received research grants from Abbott Laboratories and Boston Scientific. P.D.L. has received research grants and honoraria from Abbott Laboratories, Medtronic, and Boston Scientific. H.L. has received research funding and equipment from Siemens Healthineers and is deputy editor of Radiology: Cardiothoracic Imaging.

## Data availability

Data access statement: Individual participant de-identified data (patient demographics, MRI scan details, CIED details and parameter measurements pre and post MRI) are available upon reasonable request.

## Supplementary Material

ehab350_Supplementary_Data
