# Supplementary material for: Evidence to support magnetic resonance conditional labelling of all pacemaker and defibrillator leads in patients with cardiac implantable electronic devices
Source: Eur Heart J. 2021 Aug 26;43(26):2469–78. doi: 10.1093/eurheartj/ehab350 (PMC9259370; doi:10.1093/eurheartj/ehab350)
Supplement: ehab350_Supplementary_Data [file ehab350_supplementary_data.docx]

**Supplemental Material**

MRI in patients with cardiac implantable electronic devices: evidence to support MR-Conditional labelling of all pacemaker and defibrillator leads

Bhuva AN et al.

**Table of Contents**

[Supplemental Methods 2](#_Toc59041451)

[Consent for patients with non-MR Conditional devices 2](#_Toc59041452)

[Secondary end point analysis 2](#_Toc59041453)

[Statistics 3](#_Toc59041454)

[Supplemental Results 4](#_Toc59041455)

[References 19](#_Toc59041456)

**Index of Tables**

[Supplemental Table 1 8](#_Toc59041318)

[Supplemental Table 2 11](#_Toc59041319)

[Supplemental Table 3 12](#_Toc59041320)

[Supplemental Table 4. 13](#_Toc59041321)

Supplemental [Table 5 14](#_Toc59041322)

Supplemental [Table 6 15](#_Toc59041323)

Supplemental [Table 7 16](#_Toc59041324)

Supplemental [Table 8 17](#_Toc59041325)

Supplemental [Table 9 18](#_Toc59041326)

# Supplemental Methods

## Consent for patients with non-MR Conditional devices

MR-Conditional labelling of a complete CIED system was determined from individual manufacturer approvals in the country of MRI scanning. MRI scans were performed for patients with non-MR Conditional devices after obtaining written consent, if there was no acceptable alternative imaging modality, the results would change patient management, and the benefit was perceived to outweighed the risk of the MRI scan by the referring clinician or multidisciplinary team. Patients were excluded if they were pacing-dependent with an ICD that could not be programmed into an asynchronous mode whilst disabling anti-tachycardia therapies.[1,2]

## Secondary end point analysis

Pre-defined thresholds for significant changes attributable to MRI (outside the range of normal measurement fluctuation) were based on previously published data.[3] Thresholds were: a decrease in sensed P wave amplitude ≥ 50%; a decrease in sensed R wave amplitude ≥ 25%; an increase in capture threshold ≥ 0.5 volts (V); an absolute change in pacing lead impedance ≥ 50 Ω; an absolute change in high-voltage lead impedance ≥ 3 Ω; a decrease in battery voltage ≥ 0.04V.

Differences in lead parameter measurements between manufacturers were standardized for analysis. If a sensed amplitude was described by the pacing system analyser (PSA) as a range, or greater than a value, the lower value was recorded. Because of variability in presenting battery status between manufacturers, predicted time to ERI was recorded for all manufacturers, with voltage recorded in volts where available. For CIEDs with a clinically important change in battery status above the ERI that was not expressed as a voltage (e.g. change in categorical battery status), this was recorded as a voltage change greater than 0.04V.[3] Pacing capture thresholds were checked before and after MRI at the same pulse width, otherwise measurements were excluded from analysis.

## Statistics

Because reported event rates exceeding lead parameter cut-offs are low, confidence intervals were calculated by the Clopper-Pearson exact method.[4] Within-group changes with MRI were compared using the paired Wilcoxon signed rank test. Between-group changes were compared using the unpaired Mann-Whitney U test. Categorical variables were compared using the Chi-squared test.

To investigate whether MR-Conditional and non-MR Conditional leads demonstrated similar influences on lead-tissue interaction whilst in an MR environment, an interaction term of lead MR-Conditional labelling and the biological influence as the independent variable was used. These influences were lead and generator age (in years), thoracic MRI, presence of an ICD, and repeat MRI examinations. Repeat MRI examinations were ordered temporally, and limited to four repeat examinations per patient because of less than ten patients per group at higher repeat examinations. Separate models were used for each lead parameter, using the percentage change immediately after MRI as the dependent variable to adjust for baseline variation. Percentage changes immediately after MRI were adjusted for individual lead manufacturer. To understand the influence of repeat MRI examinations on long term trends in lead parameters, separate models were used for each lead parameter, using the absolute measurement after MRI as the dependent variable. Non-standardized correlation coefficients were used to describe the strength of associations. R^2^ was used to describe how much variation in lead parameter changes could be attributed to MR-Conditional labelling, and how much variation MR-Conditional labelling has on other lead-tissue interactions during MRI.

# Supplemental Results

| **Generators** | | | | | | | | | | | | | | |
| --- | --- | --- | --- | --- | --- | --- | --- | --- | --- | --- | --- | --- | --- | --- |
| **Abbott (n= 195)** | | | **Biotronik (n= 85)** | | | **Boston Scientific (n= 324)** | | | **Medtronic (n= 523)** | | | **Microport/Sorin/Liva Nova**  **(n= 21)** | | |
| Model | Model Number | n | Model | Model Number | n | Model | Model Number | n | Model | Model Number | n | Model | Model Number | n |
| Sustain XL | 1136 | 1 | Cylos 990 | Cylos 990 | 1 | Insignia I Plus | 1194 | 1 | Gem | 7271 | 2 | Reply 200 SR | TPM004C | 1 |
| Edurity | 1160 | 3 | Effecta | Effecta | 1 | Insignia I Ultra | 1291 | 9 | Advisa | A2DR01 | 16 | Reply 200 DR | TPM003C | 2 |
| Edurity | 1162 | 1 | Eluna 8 DR-T | Eluna 8 DR-T | 1 | Insignia I Plus | 1297 | 2 | Advisa | A3DR01 | 68 | Kora 250 DR | TPM010C | 5 |
| Edurity | 1172 | 2 | Enitra 8 HF-T QP | Enitra 8 HF-T QP | 1 | SQ-RX | 1010SQ | 4 | Advisa | A3SR01 | 1 | Reply DR | ICV1099 | 11 |
| Sentri | 1210 | 1 | Enticos 4 SR | Enticos 4 SR | 1 | Emblem | A219 | 6 | Adapta | ADDR01 | 29 | Reply | Unknown | 2 |
| Accent | 2110 | 1 | Epyra 6 SR-T | Epyra 6 SR-T | 1 | Inogen | D140 | 7 | Adapta | ADDR03 | 1 |  |  |  |
| Accent | 2124 | 2 | Etrinsa 8 SR T | Etrinsa 8 SR T | 1 | Inogen | D141 | 2 | Adapta | ADDRL1 | 43 |  |  |  |
| Sustain XL | 2136 | 2 | Evia HF-T | Evia HF-T | 1 | Inogen | D142 | 6 | Adapta | ADSR01 | 6 |  |  |  |
| Edurity | 2160 | 1 | Iforia 5 HF-T | Iforia 5 HF-T | 1 | Inogen | D143 | 2 | Syncra | C2TR01 | 2 |  |  |  |
| Edurity | 2162 | 8 | Ilesto 7 VR | Ilesto 7 VR | 1 | Dynagen | D150 | 1 | Consulta | C3TR01 | 3 |  |  |  |
| Edurity | 2172 | 7 | Intica 5 HF-T | Intica 5 HF-T | 1 | Dynagen | D151 | 1 | Consulta | C4TR01 | 2 |  |  |  |
| Accent | 2210 | 20 | Intica 5 VR-T | Intica 5 VR-T | 1 | Autogen | D174 | 10 | Viva | C5TR01 | 3 |  |  |  |
| Accent | 2224 | 8 | Itrevia 5 HF-T | Itrevia 5 HF-T | 1 | Autogen | D176 | 19 | Viruoso | D154AWG | 2 |  |  |  |
| Assurity | 2240 | 17 | Lumax 740 VR-T | Lumax 740 VR-T | 1 | Teligen 100 | E102 | 8 | Secura | D224DRG | 1 |  |  |  |
| Assurity | 2272 | 6 | Talos SR | Talos SR | 1 | Teligen 100 | E110 | 8 | Secura | D224VRC | 2 |  |  |  |
| Allure Quadra | 3140 | 1 | Epyra 8 HF-T | Epyra 8 HF-T | 2 | Energen | E140 | 1 | Maximo II | D264VRM | 1 |  |  |  |
| Anthem RF | 3210 | 2 | Itrevia 5 HF-T QP | Itrevia 5 HF-T QP | 2 | Energen | E143 | 1 | Viruoso II | D274DRG | 1 |  |  |  |
| Allure Quadra RF | 3242 | 6 | Enitra 6 DR-T | Enitra 6 DR-T | 3 | Incepta | E160 | 3 | Viruoso II | D274VRC | 1 |  |  |  |
| Quadra Allure MP | 3262 | 1 | Evia SR-T | Evia SR-T | 3 | Incepta | E162 | 4 | Maximo II | D284DRG | 1 |  |  |  |
| Quadra Allure MP | 3562 | 1 | Itrevia 7 VR-T | Itrevia 7 VR-T | 3 | Incepta | E163 | 2 | Maximo II | D284TRK | 1 |  |  |  |
| Identity Adx | 5180 | 1 | Epyra 8 DR-T | Epyra 8 DR-T | 5 | Teligen 100 | F102 | 1 | Maximo II | D284VRC | 1 |  |  |  |
| Identity XL | 5376 | 1 | Epyra 6 DR-T | Epyra 6 DR-T | 6 | Teligen 100 | F110 | 3 | Viruoso II | D294VRC | 1 |  |  |  |
| Identity Adx XL | 5386 | 2 | Estella DR-T | Estella DR-T | 8 | Teligen 100 | F111 | 2 | Protecta XT | D314DRM | 4 |  |  |  |
| Victory XL | 5816 | 1 | Itrevia 5 VR-T | Itrevia 5 VR-T | 7 | Energen | F142 | 1 | Protecta XT | D314VRG | 6 |  |  |  |
| Zephyr | 5820 | 3 | Evia DR-T | Evia DR-T | 8 | Energen | F143 | 1 | Protecta | D334DRG | 2 |  |  |  |
| Zephyr XL | 5826 | 16 | Iforia 5 DR-T | Iforia 5 DR-T | 11 | Incepta | F163 | 2 | Protecta | D334VRG | 2 |  |  |  |
| Current | 1207-36 | 1 | Itrevia 5 DR-T | Itrevia 5 DR-T | 12 | Inogen x4 | G146 | 2 | Protecta | D334VRM | 2 |  |  |  |
| Fortify | 1231-40Q | 2 |  |  |  | Inogen x4 | G148 | 4 | Protecta XT | D354DRG | 1 |  |  |  |
| Fortify | 1233-40 | 1 |  |  |  | Dynagen | G150 | 1 | Protecta XT | D354DRM | 1 |  |  |  |
| Fortify | Unknown | 1 |  |  |  | Dynagen x4 | G156 | 2 | Protecta | D364DRG | 3 |  |  |  |
| Fortify Assura | 1357-40Q | 3 |  |  |  | Autogen | G172 | 1 | Protecta | D364DRM | 2 |  |  |  |
| Ellipse | 1377-36Q | 1 |  |  |  | Autogen x4 | G177 | 3 | Protecta | D364VRM | 1 |  |  |  |
| Ellipse | 1377-36QC | 1 |  |  |  | Autogen x4 | G179 | 14 | Evera XT | DDBB1D1 | 6 |  |  |  |
| Ellipse | 1411-36C | 2 |  |  |  | Resonate x4 | G447 | 2 | Evera XT | DDBB1D4 | 3 |  |  |  |
| Current + | 2211-36 | 3 |  |  |  | Contak Renewal | H120 | 2 | Evera XT | DDBB2D1 | 4 |  |  |  |
| Fortify | 2231-40 | 1 |  |  |  | Ingenio | J173 | 4 | Evera XT | DDBB2D4 | 3 |  |  |  |
| Fortify | 2233-40Q | 1 |  |  |  | Ingenio | J174 | 4 | Evera S | DDBC3D1 | 2 |  |  |  |
| Fortify Assura | 2259-40Q | 1 |  |  |  | Ingenio MRI | J175 | 2 | Evera S | DDBC3D4 | 3 |  |  |  |
| Ellipse | 2277-36Q | 1 |  |  |  | Ingenio MRI | J176 | 3 | Evera MRI XT | DDMB1D4 | 1 |  |  |  |
| Ellipse | 2311-36Q | 9 |  |  |  | Ingenio MRI | J177 | 2 | Evera MRI XT | DDMB2D4 | 13 |  |  |  |
| Fortify Assura | 2357-40C | 1 |  |  |  | Vitalio MRI | J276 | 1 | Evera MRI XT | DDMC3D4 | 42 |  |  |  |
| Fortify Assura | 2357-40Q | 2 |  |  |  | Advantio | K063 | 1 | Protecta XT | DR314DRG | 6 |  |  |  |
| Fortify Assura | 2359-40QC | 2 |  |  |  | Ingenio | K172 | 1 | Protecta XT | DR314TRG | 3 |  |  |  |
| Ellipse | 2377-36C | 1 |  |  |  | Ingenio | K173 | 13 | Viva XT | DTBA1D1 | 2 |  |  |  |
| Ellipse | 2377-36QC | 12 |  |  |  | Essentio | L101 | 1 | Viva XT | DTBA1D4 | 4 |  |  |  |
| Ellipse | 2411-36C | 1 |  |  |  | Essentio MRI | L110 | 4 | Viva Quad XT | DTBA1QQ | 2 |  |  |  |
| Ellipse | 2411-36Q | 1 |  |  |  | Essentio MRI | L111 | 4 | Viva Quad XT | DTBA1Q1 | 2 |  |  |  |
| Promote Accel | 3215-36Q | 1 |  |  |  | Essentio MRI EL | L121 | 1 | Viva XT | DTBA2D1 | 5 |  |  |  |
| Unify | 3231-40 | 1 |  |  |  | Essentio MRI EL | L131 | 1 | Viva Quad XT | DTBA2QQ | 4 |  |  |  |
| Promote Quadra | 3239-40Q | 1 |  |  |  | Proponent MRI | L210 | 3 | Viva S | DTBB1D4 | 1 |  |  |  |
| Unify Quadra | 3249-40 | 1 |  |  |  | Proponent MRI | L211 | 18 | Brava XT | DTBC2D1 | 1 |  |  |  |
| Unify Quadra | 3249-40Q | 1 |  |  |  | Proponent MRI | L231 | 51 | Brava Quad XT | DTBC2QQ | 1 |  |  |  |
| Quadra Assura | 3265-40 | 1 |  |  |  | Accolade MRI | L301 | 3 | Claria Quad XT | DTMA2QQ | 5 |  |  |  |
| Quadra Assura | 3265-40Q | 8 |  |  |  | Accolade MRI | L311 | 1 | Amplia MRI XT | DTMB2D4 | 1 |  |  |  |
| Unify Assura | 3357-40 | 2 |  |  |  | Accolade EL | L321 | 5 | Amplia Quad XT | DTMB2QQ | 6 |  |  |  |
| Unify Assura | 3357-40C | 1 |  |  |  | Accolade MRI | L331 | 3 | Compia XT | DTMC2D1 | 1 |  |  |  |
| Unify Assura | 3357-40Q | 1 |  |  |  | Cognis 100-D | N118 | 1 | Compia MRI XT | DTMC2D4 | 1 |  |  |  |
| Unify Assura | 3361-40C | 1 |  |  |  | Cognis 100-D | N119 | 4 | Compia Quad XT | DTMC2QQ | 6 |  |  |  |
| Quadra Assura | 3365-40Q | 5 |  |  |  | Energen | N141 | 1 | Evera XR | DVBB2D1 | 1 |  |  |  |
| Quadra Assura | 3367-40Q | 1 |  |  |  | Incepta | N160 | 3 | Evera S | DVBC3D1 | 1 |  |  |  |
| Quadra Assura | 3367-40QC | 1 |  |  |  | Cognis 100-D | P107 | 2 | Evera S | DVBC3D4 | 2 |  |  |  |
| Quadra Assura MP | 3369-40C | 1 |  |  |  | Cognis 100-D | P108 | 1 | Visia AF MRI | DVFC3D4 | 1 |  |  |  |
| Quadra Assura MP | 3369-40Q | 2 |  |  |  | Energen | P143 | 1 | Evera MRI XT | DVMB2D4 | 5 |  |  |  |
| Unknown |  | 3 |  |  |  | Incepta | P162 | 1 | Evera MRI S | DVMC3D4 | 6 |  |  |  |
|  |  |  |  |  |  | Incepta | P163 | 2 | Ensura | EN1DR01 | 125 |  |  |  |
|  |  |  |  |  |  | Altura 20 | S203 | 1 | Ensura | EN1SR01 | 6 |  |  |  |
|  |  |  |  |  |  | Altura 50 | S501 | 1 | EnRhythm | P1501 | 1 |  |  |  |
|  |  |  |  |  |  | Altura 50 | S502 | 5 | Relia | REDR01 | 1 |  |  |  |
|  |  |  |  |  |  | Altura 50 | S508 | 1 | Sensia | SEDR01 | 9 |  |  |  |
|  |  |  |  |  |  | Altura 60 | S601 | 3 | Sensia | SESR01 | 4 |  |  |  |
|  |  |  |  |  |  | Altura 60 | S602 | 1 | Versa | VEDR01 | 9 |  |  |  |
|  |  |  |  |  |  | Altura 60 | S603 | 8 | Azure XT | W2DR01 | 2 |  |  |  |
|  |  |  |  |  |  | Altura 60 EL | S606 | 9 | Azure S | W3DR01 | 1 |  |  |  |
|  |  |  |  |  |  | Vitality | T177 | 2 | Percepta Quad | W4TR04 | 2 |  |  |  |
|  |  |  |  |  |  | Visionist | U225 | 1 | Solara Quad | W4TR06 | 2 |  |  |  |
|  |  |  |  |  |  | Visionist | U228 | 7 | E60A1 | E60A1 | 1 |  |  |  |
|  |  |  |  |  |  | Invive | W173 | 1 | Unknown |  | 4 |  |  |  |
|  |  |  |  |  |  | Unknown |  | 4 |  |  |  |  |  |  |

Supplemental Table 1 Cardiac implantable electronic device generator manufacturer and models implanted in patients undergoing magnetic resonance imaging. Vitratron models categorized as Medtronic. Data are for whole cohort (n=1148 MRI examinations).

| **Right Atrial Leads** | | | | | | | | | | | | | | |
| --- | --- | --- | --- | --- | --- | --- | --- | --- | --- | --- | --- | --- | --- | --- |
| **Abbott (n= 198)** | | | **Biotronik (n= 55)** | | | **Boston Scientific (n= 204)** | | | **Medtronic (n= 504)** | | | **Microport/Sorin/Liva Nova**  **(n= 5)** | | |
| Model | Model Number | n | Model | Model Number | n | Model | Model Number | n | Model | Model Number | n | Model | Model  Number | n |
| Isoflex Optim | 1944 | 8 | Siello S |  | 1 | - | 4024 | 1 | CapSureFix | 4067 | 1 | S200 | S200 AB P0179 | 1 |
| Optisense | 1999 | 3 | Safio S |  | 6 | Flextend | 4086 | 4 | CapSureFix Novus | 4076 | 32 | Beflex RF 45D | JR5CN | 4 |
| Tendril | 1188T | 1 | Setrox S |  | 3 | Flextend | 4087 | 4 | CapSure SP Novus | 4092 | 4 |  |  |  |
| Tendril LPA | 1200M | 15 | Solia JT |  | 5 | Flextend II | 4096 | 11 | CapSure SP | 4523 | 1 |  |  |  |
| Tendril DX T/TC | 1388T | 5 | Solia S |  | 39 | Flextend II | 4097 | 1 | CapSure SP | 4524 | 4 |  |  |  |
| Membrane E T/K | 1450T | 1 | Tilda JT53 |  | 1 | Flextend | Unknown | 1 | CapSureFix | 4568 | 1 |  |  |  |
| T/K | 1474T | 3 |  |  |  | Dextrus | 4135 | 8 | CapSure Sense | 4574 | 75 |  |  |  |
| Tendril SDX T/TC | 1488T | 5 |  |  |  | Dextrus | 4136 | 7 | CapSure SP Novus | 4592 | 28 |  |  |  |
| Isoflex S | 1642T | 12 |  |  |  | Fineline II EZ Sterox | 4469 | 8 | CapsureEpi | 4968 | 2 |  |  |  |
| Tendril SDX T/TC | 1688TC | 33 |  |  |  | Fineline II EZ Sterox | 4470 | 11 | TargetTip | 5028 | 1 |  |  |  |
| Optisense T/TC | 1699TC | 1 |  |  |  | Fineline II EZ Sterox AJ | 4479 | 3 | CapSureFix | 5068 | 5 |  |  |  |
| Tendril T/TC | 1782TC | 5 |  |  |  | Fineline II EZ Sterox AJ | 4480 | 15 | CapSureFix Novus | 5076 | 284 |  |  |  |
| Tendril T/TC | 1788T | 1 |  |  |  | Ingevity | 7732 | 1 | CapSureFix Novus | 5086 | 32 |  |  |  |
| Tendril T/TC | 1788TC | 1 |  |  |  | Ingevity | 7736 | 22 | CapSureFix | 5568 | 15 |  |  |  |
| Tendril ST Optim T/TC | 1882TC | 7 |  |  |  | Ingevity | 7740 | 4 | CapSure SP Novus | 5592 | 5 |  |  |  |
| Tendril ST Optim T/TC | 1888TC | 21 |  |  |  | Ingevity | 7741 | 95 | CapSure SP Novus | 5594 | 7 |  |  |  |
| Tendril STS | 2088TC | 76 |  |  |  | - | 430-05 | 2 | CapSureFix | 6940 | 1 |  |  |  |
|  |  |  |  |  |  | - | 404-01 | 1 | CapSure SP | 5524M | 4 |  |  |  |
|  |  |  |  |  |  | Unknown |  | 5 | - | IMK49JB | 2 |  |  |  |
| **Right Ventricular Leads** | | | | | | | | | | | | | | |
| **Abbott (n= 205)** | | | **Biotronik (n= 81)** | | | **Boston Scientific (n= 280)** | | | **Medtronic (n= 563)** | | | **Microport/Sorin/Liva Nova**  **(n= 6)** | | |
| Riata | 1580 | 5 | Linox Smart SD |  | 2 | Endotak Reliance S | 128 | 2 | CapSure SP | 4024 | 3 | Beflex | RF45D | 1 |
| Riata | 1581 | 2 | Plexa S |  | 1 | Endotak Endurance EZ | 154 | 3 | CapSure Sense | 4074 | 61 | S200 | S200B | 1 |
| IsoFlex Optim | 1948 | 15 | Protego S |  | 28 | Endotak Reliance | 157 | 7 | CapSureFix Novus | 4076 | 24 | Beflex | RF46D | 3 |
| Riata | 7000 | 1 | Protego SD |  | 2 | Endotak Reliance | 158 | 7 | CapSure SP Novus | 4092 | 25 |  | T44F | 1 |
| Riata | 7001 | 1 | Protego S DX |  | 2 | Endotak Reliance SG | 170 | 1 | CapSure Sense | 4574 | 1 |  |  |  |
| Riata ST | 7010 | 1 | Safio S |  | 7 | Endotak Reliance G | 175 | 2 | CapSure SP Novus | 4592 | 4 |  |  |  |
| Riata ST | 7040 | 1 | Selox ST |  | 1 | Endotak Reliance SG | 180 | 1 | CapSure Epi | 4968 | 2 |  |  |  |
| Riata ST | 7042 | 1 | Setrox S |  | 8 | Endotak Reliance SG | 181 | 4 | Target Tip | 5028 | 1 |  |  |  |
| Durata | 7120 | 10 | Siello S |  | 2 | Endotak Reliance | 184 | 1 | CapSure Z | 5034 | 1 |  |  |  |
| Durata | 7122 | 6 | Solia S |  | 23 | Endotak Reliance G | 184 | 9 | CapSure VDD II | 5038 | 1 |  |  |  |
| Durata | 7170 | 1 | Synox SX |  | 1 | Endotak Reliance G | 185 | 10 | CapSure Z Novus | 5054 | 2 |  |  |  |
| Durata | 7171 | 1 | Tilda T60 |  | 1 | Endotak Reliance 4-Site | 275 | 1 | CapSure Fix | 5068 | 3 |  |  |  |
| Durata | Unknown | 1 | TIR60BP |  | 1 | Endotak Reliance 4-Site | 276 | 1 | CapSure Fix Novus | 5076 | 184 |  |  |  |
| Tendril LPA | 1200M | 14 | Unknown |  | 2 | Endotak Reliance 4-Site Gore | 282 | 1 | CapSure Fix Novus | 5086 | 33 |  |  |  |
| Passive Plus | 1246T | 1 |  |  |  | Endotak Reliance 4-Site Gore | 285 | 5 | CapSure SP Novus | 5092 | 14 |  |  |  |
| Passive Plus | 1336T | 2 |  |  |  | Endotak Reliance 4-Site Gore | 292 | 21 | Spectrax | 5957 | 2 |  |  |  |
| Tendril DX T/TC | 1388T | 2 |  |  |  | Endotak Reliance 4-Site Gore | 293 | 6 | Sprint Fidelis | 6931 | 1 |  |  |  |
| Membrane Ex | 1450T | 2 |  |  |  | Endotak Reliance 4-Site Gore | 295 | 4 | Sprint | 6932 | 1 |  |  |  |
| Membrane EX | 1470T | 2 |  |  |  | Endotak Reliance 4-Site Gore | 296 | 4 | Sprint Quattro Secure S | 6935 | 13 |  |  |  |
| Tendril SDX T/TC | 1488T | 4 |  |  |  | Reliance 4-Front | 682 | 4 | Sprint | 6942 | 2 |  |  |  |
| IsoFlex S | 1646T | 14 |  |  |  | Reliance 4-Front | 692 | 28 | Sprint Quattro | 6944 | 5 |  |  |  |
| Tendril SDX T/TC | 1688TC | 17 |  |  |  | Reliance 4-Front | 693 | 4 | Sprint | 6945 | 2 |  |  |  |
| Tendril T/TC | 1788T | 1 |  |  |  | Reliance 4-Front | 695 | 1 | Sprint Quattro Secure | 6947 | 28 |  |  |  |
| Tendril ST Optim T/TC | 1888TC | 16 |  |  |  | Reliance 4-Front | 696 | 3 | Sprint Fidelis | 6948 | 2 |  |  |  |
| Tendril STS | 2088TC | 39 |  |  |  | Flextend | 4087 | 3 | Sprint Fidelis | 6949 | 8 |  |  |  |
| Durata | 7120Q | 14 |  |  |  | Flextend | Unknown | 1 | UK | Unknown | 1 |  |  |  |
| Durata | 7122Q | 24 |  |  |  | Flextend II | 4097 | 2 | CapSure SP | 5524M | 1 |  |  |  |
| Opitsure | LDA210Q | 4 |  |  |  | Dextrus | 4136 | 2 | Sprint Quattro Secure S | 6935M | 98 |  |  |  |
| SPL | SP01 | 2 |  |  |  | Dextrus | 4137 | 9 | Sprint Quattro | 6946M | 1 |  |  |  |
| SPL | SP02 | 1 |  |  |  | Sentra | 4271 | 1 | Sprint Quattro Secure | 6947M | 34 |  |  |  |
|  |  |  |  |  |  | Selute | 4285 | 1 | Excellence PS+ | IMK49B | 2 |  |  |  |
|  |  |  |  |  |  | Fineline II Sterox | 4456 | 12 | Unknown |  | 3 |  |  |  |
|  |  |  |  |  |  | Fineline II Sterox | 4457 | 19 |  |  |  |  |  |  |
|  |  |  |  |  |  | Fineline II Sterox | 4459 | 2 |  |  |  |  |  |  |
|  |  |  |  |  |  | Fineline II EZ Sterox | 4470 | 1 |  |  |  |  |  |  |
|  |  |  |  |  |  | Fineline II EZ Sterox | 4471 | 5 |  |  |  |  |  |  |
|  |  |  |  |  |  | EasyTrak II | 4543 | 1 |  |  |  |  |  |  |
|  |  |  |  |  |  | Rapido Advance | 7716 | 1 |  |  |  |  |  |  |
|  |  |  |  |  |  | Ingevity | 7732 | 13 |  |  |  |  |  |  |
|  |  |  |  |  |  | Ingevity | 7736 | 1 |  |  |  |  |  |  |
|  |  |  |  |  |  | Ingevity | 7740 | 1 |  |  |  |  |  |  |
|  |  |  |  |  |  | Ingevity | 7741 | 2 |  |  |  |  |  |  |
|  |  |  |  |  |  | Ingevity | 7742 | 70 |  |  |  |  |  |  |
|  |  |  |  |  |  | Ingevity | Unknown | 1 |  |  |  |  |  |  |
|  |  |  |  |  |  | - | 430-07 | 1 |  |  |  |  |  |  |
|  |  |  |  |  |  | Unknown | - | 1 |  |  |  |  |  |  |
| **Left Ventricular Leads** | | | | | | | | | | | | | | |
| **Abbott (n= 44)** | | | **Biotronik (n= 7)** | | | **Boston Scientific (n= 43)** | | | **Medtronic (n= 71)** | | | **Microport/Sorin/Liva Nova**  **(n= 0)** | | |
| Quicksite | 1056T | 1 | Corox OTW |  | 2 | Easytrak 2 | 4517 | 1 | Attain Stability Uni | 20066 | 2 |  |  |  |
| Quickflex | 1156T | 4 | Sentus OTW BP |  | 2 | Easytrak 3 | 4524 | 1 | Attain | 2188 | 1 |  |  |  |
| Quickflex µ | 1258T | 7 | Sentus OTW QP |  | 2 | Easytrak 2 | 4543 | 4 | Attain OTW | 4193 | 4 |  |  |  |
| Quartet | 1456Q | 2 | Unknown |  | 1 | Easytrak 3 | 4549 | 2 | Attain OTW | 4194 | 10 |  |  |  |
| Quartet | 1458Q | 29 |  |  |  | Acuity Steerable | 4554 | 1 | Attain Starfix | 4195 | 1 |  |  |  |
| Unknown |  | 1 |  |  |  | Acuity Steerable | 4555 | 3 | Attain Ability | 4196 | 10 |  |  |  |
|  |  |  |  |  |  | Acuity Straight | 4671 | 13 | Attain Ability Plus | 4296 | 7 |  |  |  |
|  |  |  |  |  |  | Acuity Straight 95 | 4672 | 1 | Attain Performa | 4298 | 17 |  |  |  |
|  |  |  |  |  |  | Acuity Spiral | 4674 | 5 | Attain Ability Straight | 4396 | 1 |  |  |  |
|  |  |  |  |  |  | Acuity Spiral 95 | 4675 | 1 | Attain Performa Straight | 4398 | 4 |  |  |  |
|  |  |  |  |  |  | Acuity Spiral L | 4677 | 6 | Attain Performa S | 4598 | 12 |  |  |  |
|  |  |  |  |  |  | Acuity Spiral L 95 | 4678 | 3 | CapSureEpi | 4968 | 1 |  |  |  |
|  |  |  |  |  |  | Unknown |  | 2 | Unknown |  | 1 |  |  |  |

Supplemental Table 2 Cardiac implantable electronic device lead details implanted in patients undergoing magnetic resonance imaging. Vitratron models categorized as Medtronic. Data are for whole cohort (n=1148 MRI examinations). One right atrial and one right ventricular lead did not have the manufacturer or model recorded and are not included in the table.

| **Parameter** |  | **MR-Conditional** | | | | |  | **Non-MR Conditional** | | | | |  |
| --- | --- | --- | --- | --- | --- | --- | --- | --- | --- | --- | --- | --- | --- |
|  | No of comparisons | Before | | Change (95% CI) | | *P* | No of comparisons | Before | | Change (95% CI) | | *P* | *P*  *Between- group*  *% difference* |
| **Right Atrial lead** |  |  |  |  |  |  |  |  |  |  |  |  |  |
| Amplitude/ mV | 124 | 3.40 | (2.6,4.4) | -0.1 | (0.1,-0.2) | *0.430* | 161 | 3.15 | (1.9,4.6) | -0.1 | (0.05,-0.25) | *0.289* | 0.159 |
| Threshold/V | 119 | 0.70 | (0.5,0.88) | 0.0 | (0.05,-0.15) | *0.455* | 150 | 0.75 | (0.5,1) | 0.0 | (0.1,-0.01) | *0.267* | 0.647 |
| Impedance/Ω | 124 | 516.50 | (456,622) | -10.0 | (-3.5,-17) | *0.004* | 170 | 450.00 | (399,511) | 0.5 | (5.5,-4) | *0.679* | 0.028 |
| **Right Ventricular Lead** |  |  |  |  |  |  |  |  |  |  |  |  |  |
| Amplitude/ mV | 152 | 12.00 | (8,18.9) | -0.2 | (0.1,-0.45) | *0.286* | 212 | 12.00 | (8,16) | -0.6 | (-0.35,-0.8) | *0.000* | 0.156 |
| Threshold/V | 160 | 0.70 | (0.5,0.9) | 0.0 | (0.01,-0.1) | *0.453* | 231 | 0.90 | (0.75,1.2) | 0.0 | (0.07,-0.07) | *0.904* | 0.870 |
| Impedance/Ω | 157 | 456.00 | (410,513) | -12.0 | (-7,-16.5) | *0.000* | 234 | 450.50 | (386,551) | -7.5 | (-2,-12) | *0.008* | 0.065 |
| HV Impedance/Ω | 142 | 70.00 | (60,80) | 0.4 | (0.89,-0.14) | *0.15* | 223 | 55.00 | (46,70) | 0.0 | (0.5,-0.5) | *0.816* | 0.269 |
| **Left Ventricular Lead** |  |  |  |  |  |  |  |  |  |  |  |  |  |
| Amplitude/ mV | 12 | 17.50 | (15,22) | 0.4 | (1.2,-0.33) | *0.2384* | 23 | 15.45 | (8,23) | -0.4 | (0.4,-1.35) | *0.245* | 0.692 |
| Threshold/V | 31 | 1.00 | (0.75,1.4) | 0.2 | (0.25,0) | *0.026* | 74 | 1.00 | (0.76,1.44) | 0.0 | (0.12,-0.07) | *0.547* | 0.076 |
| Impedance/Ω | 28 | 600.00 | (480,717) | -22.0 | (-13,-31.5) | *0.000* | 76 | 745.00 | (568,953) | -0.5 | (10.5,-13) | *0.886* | 0.002 |

Supplemental Table 3 Changes (post minus pre) in lead parameters for implanted cardioverter defibrillators, stratified by system MR-conditionality. The total number of MRI scans in each group was n=163 for MR-conditional systems and n=237 for legacy systems. *Abbreviations: HV= High Voltage*.

| **Parameter** |  | **Mismatched generator and lead combinations** | | | | | | |  |
| --- | --- | --- | --- | --- | --- | --- | --- | --- | --- |
|  | No of comparisons | Pre | | Post | | Change | | *P* | *P*  *vs MR-Conditional CIEDs* |
| **Right Atrial lead** |  |  |  |  |  |  |  |  |  |
| Amplitude/ mV | 57 | 3.55 | (1.8,5.2) | 3.45 | (1.6,5) | -0.2 | (0.05,-0.4) | *0.141* | *0.262* |
| Threshold/V | 54 | 0.75 | (0.5,0.95) | 0.75 | (0.5,0.98) | 0.0 | (0.1,-0.1) | *0.819* | *0.202* |
| Impedance/Ω | 60 | 513.00 | (456,622) | 494.00 | (450,604) | -16.0 | (-5,-26.5) | *0.004* | *0.337* |
|  |  |  |  |  |  |  |  |  |  |
| **Right Ventricular Lead** |  |  |  |  |  |  |  |  |  |
| Amplitude/ mV | 49 | 9.10 | (5.9,12.1) | 9.10 | (6,12) | -0.2 | (0.1,-0.55) | *0.185* | *0.540* |
| Threshold/V | 70 | 0.80 | (0.63,1.08) | 0.90 | (0.71,1.25) | 0.1 | (0.17,-0.05) | *0.347* | *0.411* |
| Impedance/Ω | 69 | 513.00 | (418,613) | 504.00 | (432,620) | -5.5 | (2.5,-15) | *0.276* | *0.534* |
|  |  |  |  |  |  |  |  |  |  |
| HV Impedance/Ω | 21 | 67.00 | (47,76) | 67.00 | (46,75) | -0.8 | (0.31,-1.84) | *0.155* | *0.109* |
|  |  |  |  |  |  |  |  |  |  |
| **Left Ventricular Lead** |  |  |  |  |  |  |  |  |  |
| Amplitude/ mV | 4 | 22.50 | (15,25) | 18.60 | (14,23) | -1.1 | (0.1,-1.7) | *0.423* | *0.565* |
| Threshold/V | 10 | 0.95 | (0.8,1) | 1.00 | (0.83,1) | 0.0 | (0.2,-0.1) | *1.000* | *0.169* |
| Impedance/Ω | 7 | 732.00 | (535,1133) | 680.50 | (557,908) | -23.9 | (15.78,-63.5) | *0.191* | *0.235* |

Supplemental Table 4 Comparison of changes in lead parameters for “mismatched” cardiac implantable electronic devices (CIEDs) immediately after MRI. “Mismatched” CIEDs are defined as MR-Conditional generator and legacy leads, or MR-Conditional components from different manufacturers. P values are for the within group change before and after MRI, and comparison of the change after MRI with the change after MRI for completely MR-Conditional CIED systems. *Abbreviations: HV= High Voltage*.

| **Parameter** |  | **MR-Conditional systems** | | | | | | |  | **Non-MR Conditional systems** | | | | | | | |
| --- | --- | --- | --- | --- | --- | --- | --- | --- | --- | --- | --- | --- | --- | --- | --- | --- | --- |
|  | No. | Before | | After | | Change  (95% CI) | | *P* | No. | Before | | After | | Change  (95% CI) | | *P* | *P*  *Between group*  *% difference* |
| **Right Atrial lead** |  |  |  |  |  |  |  |  |  |  |  |  |  |  |  |  |  |
| Amplitude/ mV | 390 | 3.40 | (2.4,5) | 3.40 | (2.4,4.9) | -0.1 | (0,-0.2) | *0.021* | 425 | 3.00 | (1.9,4.6) | 3.00 | (1.7,4.4) | -0.1 | (0,-0.2) | *0.013* | *0.261* |
| Threshold/V | 373 | 0.70 | (0.5,0.79) | 0.70 | (0.5,0.75) | -0.1 | (0,-0.11) | *0.029* | 412 | 0.75 | (0.5,0.9) | 0.75 | (0.5,0.9) | 0.0 | (0.07,0) | *0.092* | *0.074* |
| Impedance/Ω | 403 | 513.00 | (456,609) | 513.00 | (456,606) | -14.0 | (-9.5,-19) | *0.000* | 456 | 460.00 | (410,550) | 456.50 | (408,540) | -7.5 | (-4.5,-10.5) | *0.000* | *0.103* |
| **Right Ventricular Lead** | | | | | | | |  |  |  |  |  |  |  |  |  |  |
| Amplitude/ mV | 403 | 12.00 | (7.8,17.2) | 11.90 | (8,16) | -0.2 | (-0.05,-0.35) | *0.008* | 474 | 11.20 | (7,14) | 10.80 | (7,14) | -0.5 | (-0.35,-0.65) | *0.000* | *0.074* |
| Threshold/V | 460 | 0.75 | (0.5,0.95) | 0.75 | (0.5,0.9) | 0.0 | (0,-0.05) | *0.643* | 566 | 0.80 | (0.7,1) | 0.80 | (0.7,1.08) | 0.0 | (0.04,-0.02) | *0.756* | *0.711* |
| Impedance/Ω | 456 | 526.00 | (456,646) | 513.00 | (451,638) | -9.5 | (-6.5,-13) | *0.000* | 567 | 494.00 | (418,593) | 490.00 | (420,587) | -8.0 | (-5,-10.5) | *0.000* | *0.428* |
| HV impedance/Ω | 143 | 70.00 | (60,80) | 71.00 | (59,80) | 0.4 | (0.88,-0.15) | 0.*164* | 223 | 55.00 | (46,70) | 53.00 | (47,70) | 0.0 | (0.5,-0.5) | *0.816* | *0.282* |
| **Left Ventricular Lead** | | | | | | | |  |  |  |  |  |  |  |  |  |  |
| Amplitude/ mV | 18 | 17.50 | (15,23) | 16.70 | (14,23) | 0.4 | (1.6,-0.95) | *0.480* | 27 | 15.90 | (9,23) | 16.05 | (9,20) | -0.6 | (0.25,-1.35) | *0.098* | *0.553* |
| Threshold/V | 46 | 1.05 | (0.75,1.45) | 1.10 | (0.75,1.48) | 0.2 | (0.25,0.04) | *0.019* | 96 | 1.00 | (0.75,1.5) | 1.00 | (0.75,1.5) | 0.0 | (0.09,-0.1) | *0.984* | *0.029* |
| Impedance/Ω | 43 | 640.00 | (513,855) | 643.00 | (511,835) | -26.5 | (-18,-37.5) | *0.000* | 96 | 733.00 | (570,936) | 726.00 | (579,911) | -6.0 | (5,-16.5) | *0.258* | *<0.001* |

Supplemental Table 5 Changes in lead parameters when stratified by CIED system conditionality. Differences are taken as post minus pre readings. *Abbreviations: HV= High Voltage; No.= Number of lead comparisons.*

| **Patient characteristics** |  | **Long term follow-up category** | | | |  |
| --- | --- | --- | --- | --- | --- | --- |
|  |  | **Available** | | **Unavailable** | | ***P*** |
| Number of scans |  | 740 | | 309 | |  |
| Age/years |  | 69 | (57,76) | 73 | (60,80) | *0.004* |
| Male |  | 518 | (70%) | 213 | (69%) | *0.79* |
| Hospitalized inpatient |  | 48 | (6%) | 13 | (4%) | *0.31* |
| Previous MRI with an implanted cardiac device |  | 119 | (16%) | 49 | (16%) | *0.99* |
| **Indication** |  |  |  |  |  |  |
| Cardiac |  | 307 | (41%) | 128 | (41%) | *0.99* |
| Spine |  | 164 | (22%) | 102 | (33%) | *<0.001* |
| Head |  | 196 | (26%) | 79 | (26%) | *0.82* |
| Abdomen or Pelvis |  | 87 | (12%) | 35 | (11%) | *0.92* |
| Extremity or joint |  | 34 | (5%) | 9 | (3%) | *0.28* |
| Other |  | 1 | (0%) | 8 | (3%) | *<0.001* |
| **Device** |  |  |  |  |  |  |
| PPM |  | 327 | (44%) | 281 | (91%) |  |
| ICD |  | 277 | (37%) | 7 | (2%) |  |
| CRT-P |  | 24 | (3%) | 17 | (6%) |  |
| CRT-D |  | 112 | (15%) | 4 | (1%) |  |
| **Pulse generator characteristics** |  |  |  |  |  |  |
| Pacing-dependent |  | 114 | (15%) | 44 | (14%) |  |
| Legacy |  | 380 | (51%) | 111 | (36%) |  |
| **Implantation age/ years** |  |  |  |  |  |  |
| Generator |  | 2 | (1,4) | 2 | (1,4) | *0.28* |
| Number generators greater than 10 years old |  | 7 | (1%) | 4 | (1%) |  |
| Lead age |  | 3 | (1,7) | 3 | (1,6) | *0.55* |
| Oldest lead age per patient |  | 3 | (1,7) | 3 | (1,6) |  |
| Number leads greater than 10 years old |  | 183 | (25%) | 45 | (15%) |  |
| Abandoned leads |  | 33 | (4%) | 5 | (2%) | *0.04* |
| **Number of Leads (proportion non-MRI conditional)** | |  |  |  |  |  |
| RA lead |  | 616 | (23%) | 276 | (23%) |  |
| RV lead |  | 350 | (25%) | 292 | (23%) |  |
| HV lead |  | 389 | (22%) | 11 | (9%) |  |
| LV lead |  | 134 | (35%) | 20 | (15%) |  |

Supplemental Table 6 Comparison of baseline characteristics in patients with and without late follow-up. Total number of MRI scans are the number with lead parameters recorded at baseline (n=1049). *Abbreviations:* *PPM= permanent pacemaker; RA= Right Atrial; RV= Right Ventricular; HV=High Voltage; ICD= implantable cardioverter-defibrillator; LV=Left Ventricular; PPM=permanent pacemaker; MRI= magnetic resonance imaging*

| **Parameter** |  | **MR-Conditional leads** | | | | |  | **Non-MR Conditional leads** | | | | |  |
| --- | --- | --- | --- | --- | --- | --- | --- | --- | --- | --- | --- | --- | --- |
|  | **No of**  **comparisons** | **Baseline** | | **Change (95% CI)** | | ***P*** | **No of**  **comparisons** | **Baseline** | | **Change (95% CI)** | | ***P*** | ***P***  ***Between-group***  ***% difference*** |
| **Atrial lead** |  |  |  |  |  |  |  |  |  |  |  |  |  |
| Amplitude/ mV | 369 | 3.40 | (2.1,4.9) | -0.2 | (-0.05,-0.25) | *0.010* | 111 | 2.70 | (1.6,4.5) | 0.0 | (0.2,-0.15) | *0.853* | *0.472* |
| Threshold/V | 331 | 0.70 | (0.5,0.8) | 0.0 | (0.04,-0.02) | *0.789* | 101 | 0.75 | (0.6,1) | 0.0 | (0.1,-0.08) | *0.813* | *0.771* |
| Impedance/Ω | 393 | 490.50 | (437,585) | -9.0 | (-4,-13.5) | *0.000* | 123 | 450.00 | (390,570) | -5.5 | (0.5,-13) | *0.099* | *0.381* |
| **Right Ventricular Lead** |  |  |  |  |  |  |  |  |  |  |  |  |  |
| Amplitude/ mV | 396 | 12.00 | (7.9,18.3) | -0.6 | (-0.3,-0.85) | *0.000* | 116 | 10.45 | (6,12) | -0.4 | (0,-0.9) | *0.055* | *0.329* |
| Threshold/V | 440 | 0.75 | (0.6,1) | 0.0 | (0.05,0) | *0.249* | 123 | 1.00 | (0.75,1.25) | 0.0 | (0.01,-0.1) | *0.421* | *0.055* |
| Impedance/Ω | 470 | 490.00 | (419,588) | -8.5 | (-4,-12.5) | *0.000* | 150 | 530.00 | (436,648) | -6.0 | (0.5,-14) | *0.089* | *0.359* |
| HV Impedance/Ω | 114 | 65.50 | (51,77) | -0.5 | (1,-1.5) | *0.578* | 15 | 51.00 | (47,62) | -2.0 | (3,-10) | *0.462* | *0.412* |
| **Left Ventricular Lead** |  |  |  |  |  |  |  |  |  |  |  |  |  |
| Amplitude/ mV | 18 | 17.10 | (7,25) | 0.3 | (3,-1.75) | *0.7239* | 8 | 15.90 | (13,19) | 0.3 | (8.35,-7.72) | *0.929* | *0.811* |
| Threshold/V | 57 | 1.10 | (0.8,1.5) | 0.1 | (0.25,0.06) | *0.003* | 26 | 1.00 | (0.75,1.48) | 0.2 | (0.37,-0.1) | *0.157* | *0.691* |
| Impedance/Ω | 62 | 755.00 | (591,871) | -23.0 | (-4,-40.5) | *0.020* | 31 | 656.50 | (459,952) | -11.5 | (5.43,-28.46) | *0.175* | *0.314* |

Supplemental Table 7 Changes (follow-up minus baseline) in lead parameters at late follow-up. Comparisons between MR-Conditional and legacy leads are made as comparisons of percentage change to avoid baseline differences in absolute measurements. The number of lead comparisons is smaller than the number of total patients because it depends on the number of leads implanted, variable underlying rhythm, variable pulse width, and the presence of atrial fibrillation. *Abbreviations: HV= High Voltage*.

| **Parameter** |  | **MR-Conditional** | | | | **Non-MR Conditional** | | |
| --- | --- | --- | --- | --- | --- | --- | --- | --- |
|  | Pre-specified threshold |  | Number  (%) | | 95% CI | Number  (%) | | 95% CI |
| **Right Atrial lead** |  |  |  |  |  |  |  |  |
| Amplitude/ mV | -50% |  | 14 | (4%) | (2.1,6.3%) | 5 | (5%) | (14.5,10.2%) |
| Threshold/V | +0.5V |  | 7 | (2%) | (0.9,4.3%) | 5 | (5%) | (1.6,11.1%) |
| Impedance/Ω | ±50Ω |  | 72 | (18%) | (14.6,22.5%) | 19 | (15%) | (9.6,23.1%) |
| **Right Ventricular Lead** |  |  |  |  |  |  |  |  |
| Amplitude/ mV | -25% |  | 46 | (12%) | (8.6,15.2%) | 15 | (13%) | (7.4,20.4%) |
| Threshold/V | +0.5V |  | 11 | (3%) | (1.3,4.4%) | 4 | (3%) | (0.9,8.1%) |
| Impedance/Ω | ±50Ω |  | 103 | (22%) | (18.2,25.9%) | 29 | (19%) | (13.3,26.6%) |
| HV Impedance/Ω | ±3Ω |  | 73 | (64%) | (54.5,72.8%) | 9 | (60%) | (32.3,83.7%) |
| **Left Ventricular Lead** |  |  |  |  |  |  |  |  |
| Amplitude/ mV | -25% |  | 0 | (0%) | (0,18.5%) | 1 | (13%) | (31.2,52.7%) |
| Threshold/V | +0.5V |  | 6 | (11%) | (4.0,21.5%) | 3 | (12%) | (2.4,30.2%) |
| Impedance/Ω | ±50Ω |  | 31 | (50%) | (37.0,63.0%) | 6 | (19%) | (7.5,37.4%) |
|  |  |  |  |  |  |  |  |  |
| **All parameters** | **Total** |  | 142 | (10%) | (8.1,11.1%) | 43 | (7%) | (5.2,9.5%) |
|  |  |  |  |  |  |  |  |  |

Supplemental Table 8 Number of leads exceeding the secondary end-point cut-offs at late follow-up. Percentages are the absolute number divided by the total number of comparisons for that lead parameter as stated in Supplementary Table 1. The proportion of changes that were detected immediately after MRI and persistent at follow-up were 24, 32, 34, and 51% for right atrial, right ventricular, high voltage and left ventricular lead parameters respectively. The total number of leads are the number of individual leads meeting at least one end-point, and so is less than the total of each individual parameter.The confidence limits are calculated using the Clopper and Pearson Exact Method. *Abbreviations: CI= confidence interval; HV=High Voltage.*

| **Dependent variable** | **Lead age** | **Generator age** | **ICD vs pacemaker** | **Thoracic vs non-thoracic examination** | **Repeat imaging** | **Lead**  **Manufacturer** | **Interaction with MR-Conditionality** |
| --- | --- | --- | --- | --- | --- | --- | --- |
| *Right Atrial lead* | | | | | | | |
| Sensed amplitude | No association | No association | No association | No association | No association | No association | No association |
| Capture threshold | No association | No association | No association | No association | Between MRI:  0.04 (0.002,0.08)  p=0.039 | No association | No association |
| Impedance | No association | No association | -1.4 (-0.6,-2.3)  p<0.001 | No association | No association | -1.4,4.6  p<0.001 | Thoracic MRI:  -2.5 (0.5,4.6)  p=0.016 |
| *Right Ventricular lead* | | | | | | | |
| Sensed amplitude | No association | No association | No association | No association |  | No association | No association |
| Capture threshold | No association | No association | No association | No association | Between MRI:  0.05 (0.0007,0.1)  p=0.047 | No association | No association |
| Impedance | No association | No association | No association | No association | No association | -2.1,1.6  p=0.045 | No association |
| HV Impedance | No association | No association | No association | No association | No association | No association | No association |
| *Left Ventricular lead* | | | | | | | |
| Sensed amplitude | No association | No association | No association | No association | No association | No association | No association |
| Capture threshold | No association | No association | No association | 5.5 (0.37,10.7)  p=0.036 | No association | No association | No association |
| Impedance | 0.3 (0.01,0.6) p=0.042 | No association | -2.6 (-0.5,-4.7)  p=0.017 | No association | After MRI:  1.3 (0.4,2.2)  p=0.006 | No association | Thoracic MRI:  -6.3 (2.4, 10.2)  p=0.0018 |
|  |  |  |  |  |  |  |  |

Supplemental Table 9 Associations with lead parameter changes. Associations are non-standardized and represent the percentage change immediately after MRI with 95% confidence interval. For repeat scans over time, significant associations with the absolute change in lead parameter with each repeat MRI (‘Between MRI’) are also presented. Lead age and generator age was measured in years. Association with manufacturer is presented as range of variability. For all devices, older LV leads (but not other leads or generators) were associated with a greater rise in impedance after MRI but with no difference in other parameters. ICDs were associated with a greater fall in RA and LV lead impedances after MRI than pacemakers, but no difference in other parameter changes. Repeat MRI examinations were associated with a slightly greater rise in LV impedance immediately after MRI, and a small rise in RA and RV lead thresholds between repeat examinations. Thoracic MRI examinations were associated with a slightly greater rise in LV lead capture threshold after MRI than non-thoracic examinations. This only partly explained the greater rise in LV lead capture threshold change after MRI in MR-Conditional than non-MR Conditional leads. The reductions in RA and RV lead impedances varied slightly between lead manufacturers. MR-Conditional leads demonstrated a slightly greater decrease in right atrial and left ventricular lead impedances than non-MR Conditional leads immediately after thoracic MRI examinations. The greater decrease in LV lead impedance observed in MR-Conditional leads than non-MR Conditional leads was not attributable to LV lead manufacturer. All differences were small and explained only a small amount of total variation (1% and 10% respectively). *Abbreviations: HV= High Voltage; ICD= Implantable cardioverter-defibrillator.*

# References

1. Nazarian S, Hansford R, Rahsepar AA, Weltin V, McVeigh D, Gucuk Ipek E, Kwan A, Berger RD, Calkins H, Lardo AC, Kraut MA, Kamel IR, Zimmerman SL, Halperin HR. Safety of Magnetic Resonance Imaging in Patients with Cardiac Devices. N Engl J Med. 2017 Dec 28;377(26):2555–64.

2. Russo RJ, Costa HS, Silva PD, Anderson JL, Arshad A, Biederman RWW, Boyle NG, Frabizzio J V., Birgersdotter-Green U, Higgins SL, Lampert R, Machado CE, Martin ET, Rivard AL, Rubenstein JC, Schaerf RHM, Schwartz JD, Shah DJ, Tomassoni GF, Tominaga GT, Tonkin AE, Uretsky S, Wolff SD. Assessing the Risks Associated with MRI in Patients with a Pacemaker or Defibrillator. N Engl J Med. 2017 Feb 23;376(8):755–64.

3. Cohen JD, Costa HS, Russo RJ. Determining the Risks of Magnetic Resonance Imaging at 1 . 5 Tesla for Patients With Pacemakers and Implantable Cardioverter Defibrillators. AJC. 2009;110(11):1631–6.

4. Tobi H, van den Berg PB, de Jong-van den Berg LTW. Small proportions: What to report for confidence intervals? Pharmacoepidemiol Drug Saf. 2005;14(4):239–47.
